# High Hydrostatic Pressure Induced Changes in the Physicochemical and Functional Properties of Milk and Dairy Products: A Review

**DOI:** 10.3390/foods10081867

**Published:** 2021-08-12

**Authors:** Sergio O. Serna-Hernandez, Zamantha Escobedo-Avellaneda, Rebeca García-García, Magdalena de Jesús Rostro-Alanis, Jorge Welti-Chanes

**Affiliations:** Tecnologico de Monterrey, Escuela de Ingeniería y Ciencias, Eugenio Garza Sada 2501, Monterrey 64700, NL, Mexico; sergio_serna3@hotmail.com (S.O.S.-H.); zamantha.avellaneda@tec.mx (Z.E.-A.); rebeca.garcia.garcia@tec.mx (R.G.-G.); magda.rostro@tec.mx (M.d.J.R.-A.)

**Keywords:** high-pressure processing, milk, physicochemical properties, functional properties, dairy products

## Abstract

High-pressure processing (HPP) is a nonthermal technology used for food preservation capable of generating pasteurized milk products. There is much information regarding the inactivation of microorganisms in milk by HPP, and it has been suggested that 600 MPa for 5 min is adequate to reduce the number of log cycles by 5–7, resulting in safe products comparable to traditionally pasteurized ones. However, there are many implications regarding physicochemical and functional properties. This review explores the potential of HPP to preserve milk, focusing on the changes in milk components such as lipids, casein, whey proteins, and minerals, and the impact on their functional and physicochemical properties, including pH, color, turbidity, emulsion stability, rheological behavior, and sensory properties. Additionally, the effects of these changes on the elaboration of dairy products such as cheese, cream, and buttermilk are explored.

## 1. Introduction

In recent years, consumers have demanded more natural food products that maintain the nutritional quality of the original product, are free from preservatives or other additives, and are safe to consume [1]. As a response, the food industry has explored new alternatives and novel processes to generate food that meets consumer demands. Traditionally, milk and other products are preserved by thermal treatments (sterilization or pasteurization) to reduce microbial loads to safe levels, and to prolong their shelf life [2]. High hydrostatic pressure is a nonthermal processing method that is applied as an alternative to traditional thermal processes to ensure food safety while reducing quality loss in the treated products [3]. This process involves the use of extremely high isostatic pressures—usually between 100 and 1000 MPa—for specific short time periods [4].

High-pressure processing (HPP) is capable of inactivating microorganisms and specific enzymes at room temperature, thereby prolonging a product’s shelf life and guaranteeing its safety upon consumption [1]. Due to its minimal effects on the sensory and nutritional characteristics of the final products, HPP commonly generates foods with better acceptability and enhanced sensory properties, as quality is maintained during processing—especially when compared to their thermally treated counterparts [5]. Raw milk is a highly nutritious food—an ideal environment for the proliferation of spoilage and pathogenic microorganisms; however, when subjected to HPP, these microorganisms can be inactivated [6]. In addition to microbial inactivation, different changes are also present in milk subjected to HPP. Components such as casein, whey proteins, and fat globules can be altered. Additionally, milk’s physicochemical and functional characteristics can also be modified and altered; however, the type and extent of these changes depend mainly on the composition of the milk, the pressure, and the holding time levels, and are lower compared with thermal treatment [4].

In general, HPP is carried out in three distinct steps: the come-up time (CUT), which is the time required to reach the desired pressure intensity; the holding time, for which the desired pressure intensity is maintained; and, finally, the pressure release depressurization. HPP technology follows two important operating principles: Le Chatelier’s principle, and the isostatic principle [1,7]. Le Chatelier’s principle posits that any system under equilibrium that is subjected to pressure will adapt the molecular configuration, chemical interactions, and reactions that yield the smallest volume [1,8]. The isostatic principle explains that whenever pressure is applied to a system, it is transmitted equally and instantaneously throughout the system and food product, regardless of size or shape [1,9].

Abundant information on the effects of HPP on milk microorganisms has been reported, although not much exists regarding the changes in the physicochemical and functional properties of milk. Thus, this review aims to highlight the most recent scientific breakthroughs regarding bovine milk and other dairy products processed with HPP, and the effects that these treatments have on the physicochemical and functional properties of the products.

## 2. Microbial Inactivation in Milk with HPP

Raw milk naturally contains different microorganisms. Some pathogens commonly found are *Listeria monocytogenes,* *Salmonella*, *Escherichia coli*, *Shigella*, and *Staphylococcus aureus*, alongside spoilage bacteria [3]. The utilization of HPP in raw milk, at ambient temperature or lower and with varying pressure intensities, has proven to be an efficient alternative to thermal pasteurization [1,10]. However, because of the presence of vegetative spores, a sterilization process cannot be achieved for milk by utilizing high pressure exclusively; heat or other preservation factors need to be used in conjunction with HPP [6,11].

The application of intense heat causes important changes in some components and physical characteristics of milk—most notably, the visual color and taste of pasteurized milk differs from its raw counterpart [12]. Many authors explain how recent advances in high-pressure technology yield milk that is comparable in terms of safety and shelf life to pasteurized milk, because of the inactivation of foodborne microorganisms [13,14,15]. This is the main and most important objective of HPP, which is the reason most of the available information centers around the effects of HPP on different microorganisms, and how to achieve and obtain a safe and stable product. In recent years, the perspective has shifted, and the effects of high-pressure-treated milk on physicochemical and functional properties, and their influence on dairy products, have become more thoroughly explored.

The resistance to high pressure of pathogenic bacteria and spoilage microorganisms in raw milk depends on multiple factors, including the pressure level, holding time, temperature, milk composition, and growth phase [10,16]. The mechanisms of microbial inactivation by HPP are related to damage to the cell wall, cell membrane, genetic material, and cytoplasmic components—which, in turn, prevent and lower their recovery and growth rates [3]. A summary of the most studied microorganisms in milk and their pressure resistance can be found in Table 1. As observed, many studies have demonstrated that high-pressure treatments, without the aid of heat, can achieve a microbial population reduction of 5–7 logarithmic cycles of the most common microorganisms found in milk, such as *E. coli*, *L. monocytogenes*, *Salmonella* spp., and others. Previously, it was believed that subjecting milk to pressure intensities in the range of 300–600 MPa was an effective alternative method to traditional pasteurization [17]. However, enough scientific evidence has been generated throughout the years to prove that HPP is a suitable alternative to traditional pasteurization. It has been stated that treating milk at 600 MPa for 5 min achieves comparable results to those of heat treatment regarding microbial inactivation through logarithmic reduction, obtaining safe milk products with extended shelf life [13,15]. That being said, additional studies and information regarding the effects of HPP on microorganisms such as *Staphylococcus aureus*, *Coxiella burnetii*, *Mycobacterium*, and others that can also be present in raw milk are required in order to generate a wider perspective, and more accurately determine the safety levels of treated milk.

## 3. Effects of HPP on Milk Components

### 3.1. Lipids

Triglycerides are the main fraction of milk lipids, accounting for up to 98% of the total concentration; diglycerides, monoglycerides, cholesterol, cholesterol esters, and free fatty acids make up most of the reminder of milk lipids [28]. During processing, milk is standardized to have a specific fat content before commercialization; whole milk is considered to have around 3.25% fat, while skim or non-fat milk is relatively fat-free [29]. Reduced-fat milk and low-fat milk that contain 2% and 1% fat, respectively, are other options of commercial milk [29]. The effects of high-pressure processing on milk lipids have been studied: moderate and more intense pressure levels (250, 450, 550, 700, 800, and 900 MPa, for 5 min each) have been shown not to significantly change the quantity of triglycerides in milk; the concentration of diglycerides, monoglycerides, and free fatty acids also remains statistically similar [30]. Additionally, it has been reported that intense-pressure treatment does not produce significant changes in polar lipid content and distribution, including phospholipids [30]. A study [31] about the effect of pulsed HPP on whole milk free fatty acid composition showed that when milk is subjected to two pulses of 600 MPa/1.5 min and 2.5 min, the amount of short-chain saturated fatty acids decreases, while that of medium-chain fatty acids slightly increases. Treatment at 600 MPa for 5 min in a single cycle showed an increase in short- and medium-chain fatty acids and a decrease in long-chain fatty acids.

In milk, the lipid content is distributed in fat globules, which are surrounded by a complex mixture of proteins, enzymes, phospholipids, triglycerides, and other compounds that together form a membrane known as the milk fat globule membrane, or MFGM [32]. HPP has been shown to alter the size of fat globules and the composition of the MFGM. Pressure can slightly affect the size of fat globules; however, temperature parameters have a greater impact on size; processing milk at temperatures higher than 25 °C yields smaller fat globules, while the opposite is expected when using colder conditions [1,33,34]. Ye et al. [34] observed that whole milk subjected to 100–800 MPa promoted β-lactoglobulin (β-lg) association to the MFGM proteins through sulfhydryl disulfide interactions; increased association was promoted at higher pressures; similarly, κ-caseins and α-lactalbumin (α-la) can associate in lower quantities to the MFGM through the same interaction at pressures higher than 500 and 700 MPa, respectively. These changes and alterations to the MFGM’s composition modify its structure, stability, and integrity, as well as the milk’s properties, such as its emulsion capacity [33].

Regarding milk fat globule size, in a study [34] in which milk was subjected to different treatment conditions from 100 to 800 MPa, in 100 MPa increments for 30 min each, the average fat globule size was not affected when dispersed in buffer. In the previous study, treated milk samples were dispersed in either SDS/EDTA buffer or water to correctly evaluate their diameters. Fresh milk dispersed in buffer samples had an average particle diameter of 1.13 μm, which remained statistically similar after the eight different high-pressure treatments. Samples dispersed in water behaved differently, as an increase in diameter size was observed up to the 700 MPa treatment, whereas it remained constant with more intense treatments; this was attributed to the disruption of casein micelles, which reduced their contribution to the light-scattering effect. Similar results were observed in a more recent study [35] on caprine raw milk treated at 200, 300, 400, and 500 MPa for 10 min at 20 °C and stored at 4 °C for 14 days. Immediately after treatment, all samples had statistically similar fat globule size diameters when compared to their raw milk counterparts; however, a dramatic increase in their size was observed after the 14-day storage for the untreated, 200, and 300 MPa samples. The mean diameters for the 400 and 500 MPa samples remained statistically the same as for the samples observed immediately after treatment. The authors determined that the fat globule size of milk subjected to 400 MPa or more remained unchanged even after cold storage. With the limited available information, the effect of HPP on the particle size of milk fat globules has been debated; Garcia-Amezquita et al. [36] reported an increase in average diameter after treatments at 400 MPa for 15 and 20 min and 500 MPa for 5 and 10 min because of flocculation; meanwhile, as previously mentioned, other studies state that particle size could remain unaffected [35]. Additional information and knowledge are required to determine the specific interactions and behavior of fat globules under high pressure, and how these affect them.

**Figure 1:**
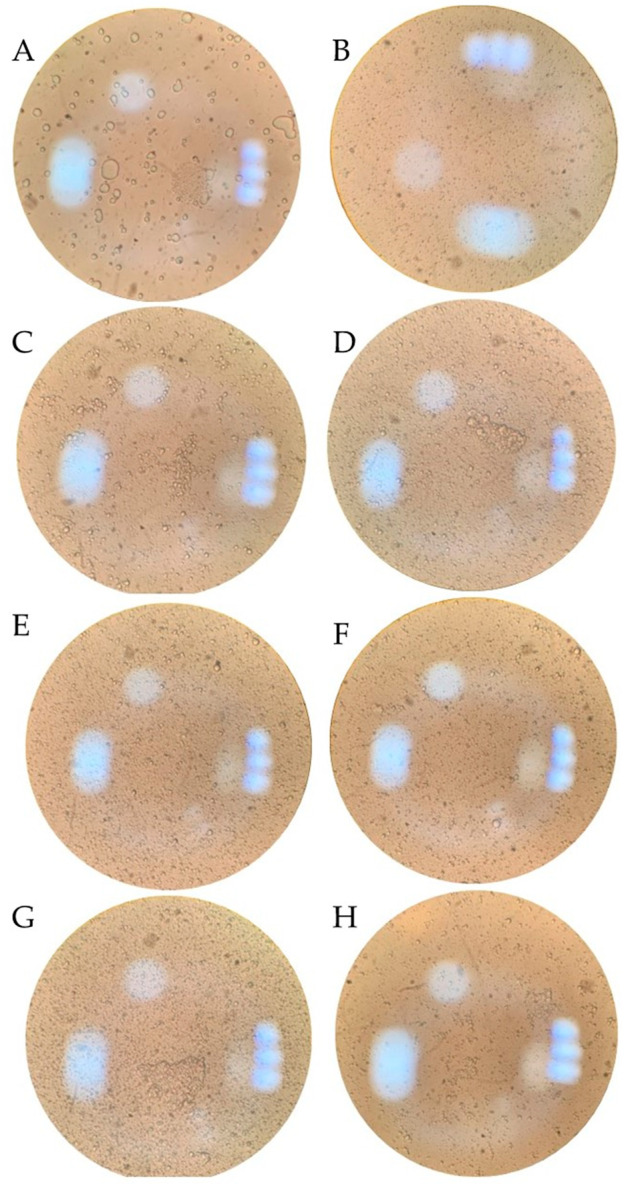
Optical 100× light microscope images of (**A**) raw milk, (**B**) homogenized (HM) ultra-pasteurized milk, (**C**) HM 400 MPa, 5 min-treated milk, (**D**) HM 400 MPa, 10 min-treated milk, (**E**) HM, 500 MPa, 5 min-treated milk, (**F**) HM 500 MPa, 10 min-treated milk, (**G**) HM 600 MPa, 5 min-treated milk and (**H**) HM 600 MPa, 10 min-treated milk.

Considering the limited amount of available information regarding HPP and its effects on milk fat globules, a preliminary visual analysis of raw milk samples was conducted in our laboratory. A variety of raw and homogenized high-pressure-treated milk samples were observed via optical light microscope, followed by visual analysis and interpretation. As seen in Figure 1, the sizes of fat globules between raw and homogenized milk are drastically different; the non-homogenized sample presented globules bigger in size, as expected. Regarding high-pressure-treated samples, similar patterns of globule distribution and size in the raw homogenized milk samples and those treated at 500 and 600 MPa were observed. The 400-MPa-treated samples presented fat globule agglomeration, and several globules were bigger in size, similar to those in raw, non-homogenized milk. These discrepancies between samples treated at different pressure intensities could lead to the separation of fat and a break in the emulsion stability, especially in those that are not subjected to homogenization, or are treated at mild pressure intensities. A commercial example milk with similar characteristics is Made by Cow—an Australian brand that offers “cold-pressed” milk subjected to HPP; they declare that their dairy products are not homogenized, and that consumers should expect a cream layer on top of the product composed of fat particles [37]. Regarding milk fat globules, new information about the specific effects of HPP on their size, structure, and relationship with emulsion stability need to be generated. Additionally, the effects of high-pressure treatment on homogenized and non-homogenized samples could differ; specific information regarding the effects of independently subjecting milk to HPP is required, and the countereffects of homogenizing samples prior to processing could also be explored. This is especially relevant today, as the focus of studies on high pressure and milk has been on microbiology, and additional nutrients and compounds, such as proteins.

As mentioned previously, HPP itself has been shown to have very little to no effect on the lipid composition of milk; however, when it is processed at a mild pressure (250 MPa) without the aid of heat, enzymatic lipolysis is promoted. Lipolysis is caused by the enzyme lipoprotein lipase, which can be naturally present in raw milk or generated by bacteria during its storage. Lipolysis causes undesired flavors and aromas because of the release of free fatty acids, as well as the generation of mono- and diglycerides and additional volatile compounds [38]. As previously mentioned, the effects of high pressure on milk lipids and fat globules need to be more deeply studied, especially because these changes have been related to changes in quality regarding emulsion stability in the final product.

### 3.2. Proteins

Milk proteins can be divided into two groups: whey proteins and caseins, which represent approximately 20% and 80% of the total protein content, respectively [39]. Proteins are composed of a specific sequence of amino acids and stabilized by covalent bonds, electrostatic interactions, hydrogen bonds, disulfide bonds, hydrophobic interactions, and more [10,40]. These bonds have different susceptibilities to HPP; the weaker bonds are hydrogen and disulfide bonds; hydrophobic and electrostatic interactions are greatly affected, whereas covalent bonds remain intact after pressurization but thermal treatments greatly disrupt them [4,10,11]. The proteins are arranged in four different sequential levels—primary, secondary, tertiary, and quaternary structures—which are folded differently, and have distinct types of bonds and interactions. Protein denaturation, which can be caused by HPP, alters the native structure of proteins by causing unfolding and damaging different bonds and interactions; these changes in milk proteins also cause alterations to functionality and properties [10,40]. The protein structures that are widely affected by HPP are the quaternary, tertiary, and secondary structures, because of the presence of weaker types of bonding; primary structures are virtually unaffected because of the presence of covalent bonds [10].

Casein micelles and whey proteins are two of the most frequently studied components in milk, because of their nutritional properties, functionalities, and characteristics, such as color and turbidity, rheological properties, pH, and emulsion stability. Regarding their structure, composition, and size, these proteins have been extensively studied, and the changes caused by different processing methods, including high pressure, are relevant because of the alterations to the specific properties of milk and dairy products, including hydration and acidification, among others [41,42].

Caseins are hydrophobic proteins, and possess a high charge; there are four different types: α_s1_-, α_s2_-, β-, and κ-caseins. These different caseins can bind together in the presence of calcium phosphate to generate a colloidal aggregate known as a casein micelle, which has a diameter in the range of 50 − 300 nm, is composed of approximately 6% inorganic matter in dry weight, and is highly hydrated [39,41,42]. The exact composition and structure of casein micelles is disputed and under debate, for which they have been extensively studied. Bhat et al. [41] summarized three distinct models for their classification: the coat–core model, sub-micellar model, and internal structure model; the first model explores how the outer coating and internal structure of micelles are composed differently, the second model dictates how rough spherical subunits make up the casein micelles, and the third model indicates the process by which caseins can aggregate differently.

The most prominent whey proteins are β-lactoglobulin (β-lg), α-lactalbumin (α-la) and, to a lesser extent, bovine serum albumin (BSA), immunoglobulins (IG), bovine lactoferrin (BLF), bovine lactoperoxidase (LP), and minor amounts of glycomacropeptide (GMP); these proteins are soluble at a pH of 4.6 [39,43]. These globular proteins have a predominant helical structure, which can be partially unfolded during milk processing, causing denaturation [44]. Furthermore, enzymes are also an important fraction of the proteins in milk, and are impacted by high pressure. From a technological standpoint, enzymes are used to determine milk quality regarding deterioration and preservation, indicators for pasteurization, detection of mastitis infection, and antimicrobial activity [45].

#### 3.2.1. Casein Micelles

**Figure 2:**
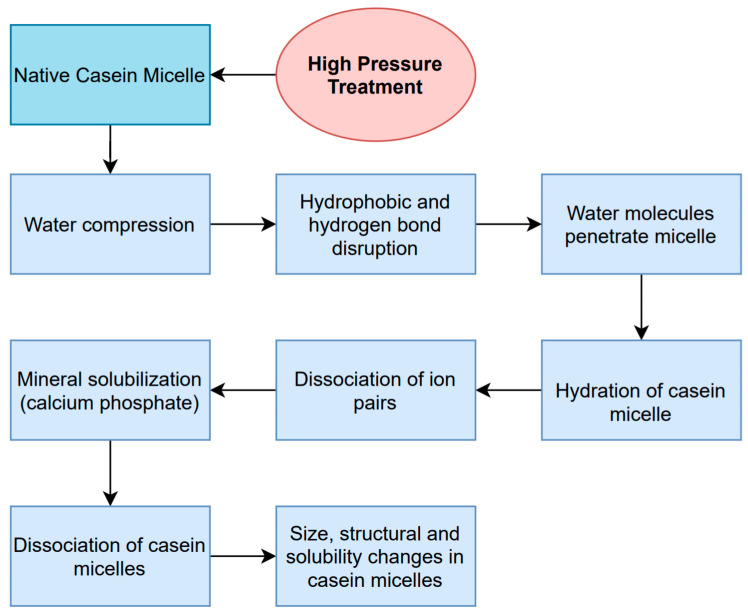
Sequence of effects regarding the denaturation of casein micelles by HPP, [7,10,39,42,47,48].

Casein micelles undergo structural changes in their size, composition, and hydration during HPP treatments; however, the specific changes, and their extent, depend on the treatment conditions the milk was subjected to. During HPP, water is compressed, which causes a disruption in the hydrophobic bonding of the casein micelles’ components; this changes the light transmission properties of milk, and minerals are solubilized—mainly micellar calcium phosphate [7,10,39,42,46,47,48]. Mineral solubility and ionization are favored under high-pressure conditions; the compacted water molecules penetrate and hydrate the molecule, causing a dissociation of ion pairs that leads to a release of calcium phosphate and physical changes in the casein micelles [10,39,47]. The changes in mineral solubility, alongside additional internal and external factors such as the presence of denatured whey proteins, temperature, pH, and milk composition, cause disassociations of casein micelles; however, reassociation is possible during storage, depending on the level of pressure applied [39,47]. A diagram and summary of the process and changes occurring to casein micelles is depicted in Figure 2. Milk subjected to high-pressure treatments undergoes two important changes: firstly, an increase in the negative charge of the molecule due to the association of denatured β-Lg, and secondly, the disruption of the micelle into the four different casein molecules; these changes increase the molecules’ hydration and solubility [10].

The size of casein micelles is greatly affected by HPP; the extent and type of the changes are determined by the treatment conditions. Pressures greater than 300 MPa cause an irreversible decrease in casein micelle size, attributed to micelle fragmentation. Milder pressures of approximately 250 MPa may increase the size of micelles because of the interaction between denatured β-Lg and κ-casein; however, these changes can be reversible [10,39,49]. Temperature and pH also contribute to micelle size changes during processing; temperature enhances size increments alongside higher pH levels [10,39,49]. Table 2 summarizes some reported changes in the size of casein micelles at different treatment conditions. In general, pressure treatments lower than 200 MPa have no effect on particle size; however, incrementing the pressure intensity tends to yield smaller micelles. Nevertheless, applying the most intense treatments eventually leads to an irreversible decrease in their size. These changes are mainly caused by the intensity of pressure treatment, whereas treatment time and temperature do not impact changes in size significantly.

A study [13] performed with whole milk processed at 600 MPa, in which casein size was analyzed with Zetasizer, showed that low-temperature low-time (LTLT), high-temperature short-time (HTST), and HPP samples had z-values of 222, 247, and 140 nm, respectively, concluding that casein particle size was significantly smaller in the high-pressure-treated samples when compared to heat-treated milk. The previous results indicate that the changes to casein micelle size are exacerbated by intense pressure treatment; temperature did not have as significant an impact even when two different conditions were analyzed. Similarly, Iturmendi et al. [51] studied the effects of subjecting 4 and 8% micellar casein concentrates (MCCs) to 300, 450, and 600 MPa for 5 min on casein size; the results were compared to untreated control samples. It was found that for the 4% concentrate the control-, 300-, 450-, and 600-MPa-treated samples had a z-average of 186, 167, 129, and 101 nm, respectively, and for the 8% concentrate had z-values of 193, 185, 162, and 167 nm, respectively. In general, size reduction increased alongside increasing pressure treatment and intensity. These authors also attributed loss of brightness and an increase in the transparency of the samples to the decrease in casein particle size; additionally, the samples subjected to 600 MPa revealed that the particles reaggregated; however, this change was more prevalent for the samples with lower micellar casein concentration, because of the increased presence of micellar calcium phosphate.

**Figure 3:**
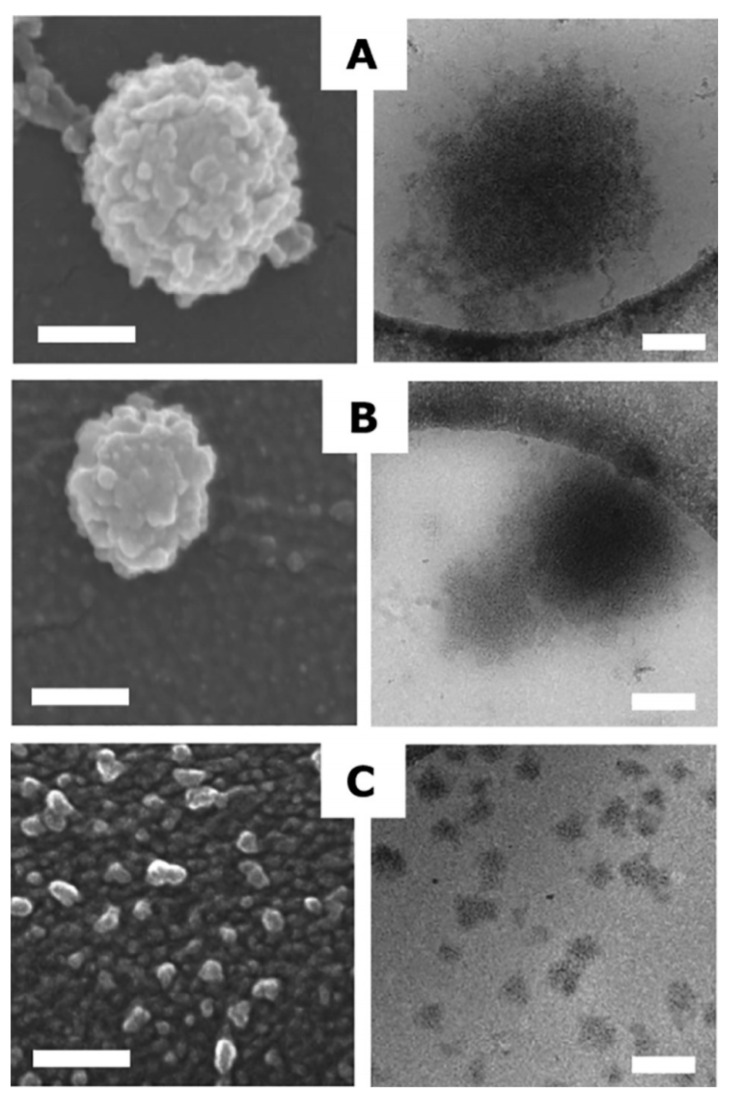
SEM (**left**) and cryo-transmission electron microscopy (**right**) micrographs of reformed casein micelles, untreated (**A**), sonicated for 30 min (**B**) and after a HPP of 500 MPa for 10 min (**C**). Scale: 100 nm [48]. Reproduced with permission from Yacine Hemar, Cheng Xu, Sinong Wu, Muthupandian Ashokkumar, Size reduction of “reformed casein micelles” by high-power ultrasound and high hydrostatic pressure; published by Elsevier, 2020.

**Figure 4:**
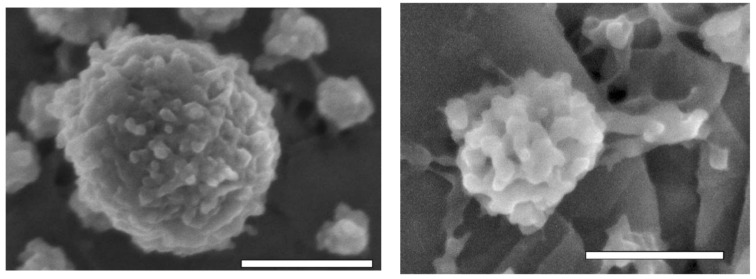
SEM micrographs of intact (**left**) and dissociated (**right**) casein micelles. Scale: 200 and 100 nm, respectively [49]. Reproduced with permission from Douglas G. Dalgleish, Paul A. Spagnuolo, H. Douglas Goff, A possible structure of the casein micelle based on high-resolution field-emission scanning electronmicroscopy; published by Elsevier, 2004.

**Figure 5:**
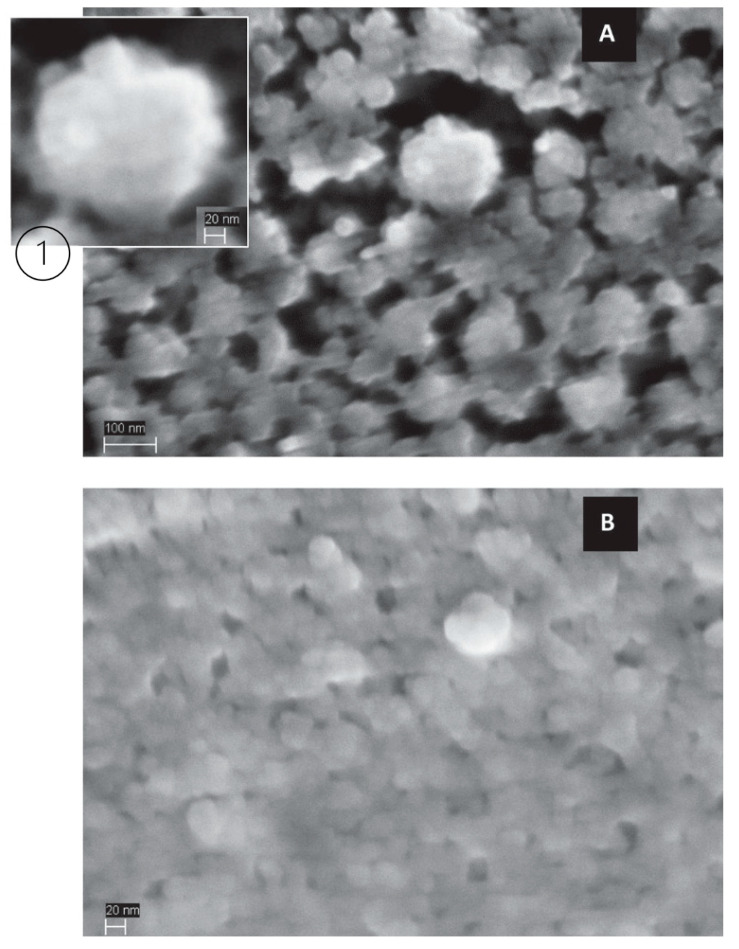

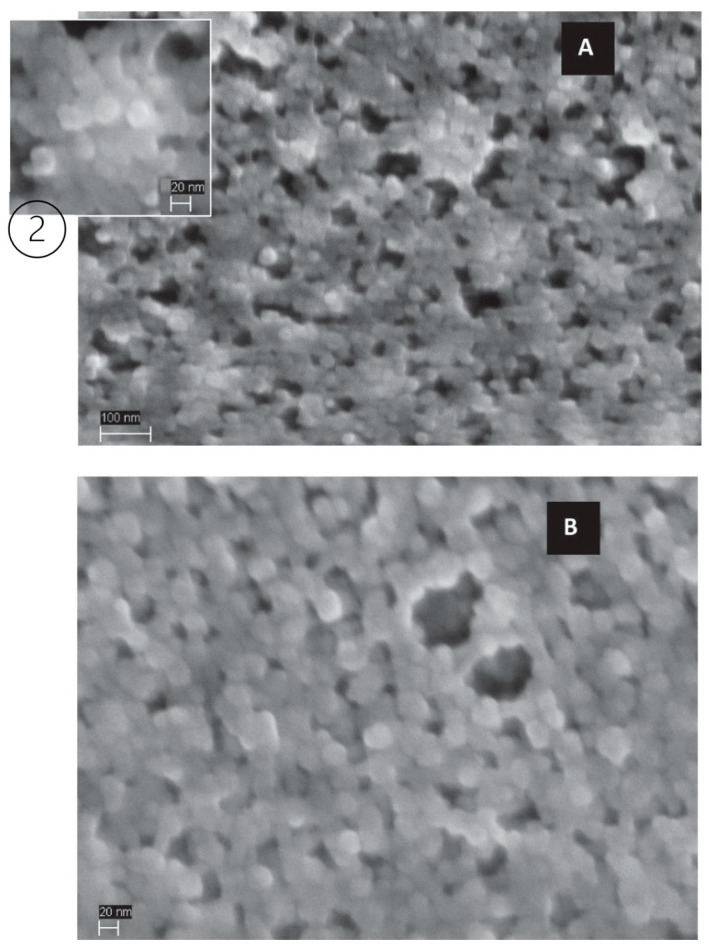
SEM images of 10% micellar casein concentrates (**1**) and 10% milk protein concentrates (**2**). Untreated samples (**A**) and 450-MPa-treated samples (**B**) [52]. Reproduced with permission from LeeCadesky, MarkusWalkling-Ribeiro, Kyle T.Kriner, Mukund V.Karwe, and Carmen I.Moraru, Structural changes induced by high-pressure processing in micellar casein and milk protein concentrates; published by Elsevier, 2017.

A commonly used study to visually evaluate casein micelle surface structure, shape, and size is electron microscopy. Scanning electron microscopy (SEM) yields different images or micrographs that help to determine differences in casein micelles. The microstructure, shape, and size of casein micelles’ micrographs has been observed in pressure-treated milk samples and compared to their untreated counterparts, revealing changes and alternations in these parameters; different examples of casein micelles are shown in Figure 3, Figure 4 and Figure 5 [48,49,52].

In Figure 3, it can be observed that after the application of 500 MPa, casein micelles had an average size of 20 nm, while for the untreated samples it was 200 nm [48]. In Figure 4, casein micelles with rough spherical shapes are observed, and casein subunits are also depicted at the micelles’ surface; moreover, possible dissociated casein micelle fragments can be observed in the backgrounds of both images; the rightmost image also depicts a smaller, dissociated casein micelle, and the presence of links to other fragments [49]. In Figure 5, untreated casein and protein concentrates show nearly perfect spherical shapes, with an average diameter of 200 nm; after the 450-MPa treatment, the spherical shape became irregular, structural integrity was lost alongside a diminution in size, and a network of 20-nm aggregates was formed [52]. As observed and explained, high-pressure treatments severely affect and change the size and structure of casein micelles; these changes have been determined as the cause of additional effects on milk properties, such as changes in color and stability, rheological properties, pH, and more.

#### 3.2.2. Whey Proteins

**Figure 6:**
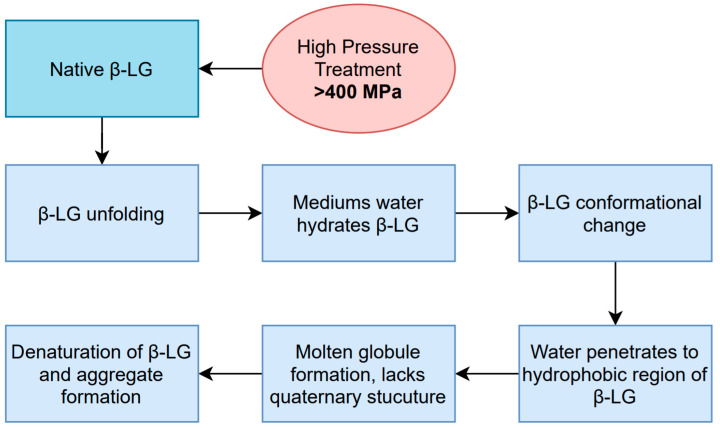
β-LG denaturation by HPP [47].

Milk proteins are affected differently by high-pressure treatments. As was previously mentioned, the primary structure of proteins is mainly composed of covalent bonds, which are virtually unaffected by HPP; however, their secondary and tertiary structures have hydrophobic, electrostatic interactions and hydrogen bonds, which can be disrupted by this process. This means that whey proteins are more susceptible to unfolding or denaturation [47]. The two most abundant whey proteins are α-lactalbumin (α-LA) and β-lactoglobulin (β-LG); α-LA is more stable and resistant to HPP—it has a more rigid structure attributed to four intramolecular disulfide bonds and a lack of thiol groups—whereas β-LG is more susceptible to HPP: its structure is characterized by two intramolecular disulfide bonds and one free thiol group [1,39,47]. The sensitivity to pressure of β-LG can be explained by a denaturation model; this process starts with a reversible unfolding caused by pressure, which allows the water molecules in the medium to penetrate the hydrophobic regions hydrating the molecule, followed by a change in the proteins’ conformation similar to a molten globule and, finally, the denaturation forms aggregates [47]. This process is summarized in Figure 6.

Immunoglobulins (IGs) and bovine serum albumin (BSA) are whey proteins present in milk in smaller concentrations. IGs are relatively sensitive to HPP; however, BSA is believed to be more pressure resistant because of its structure, which has 17 desulfated bonds and a high quantity of α-helices [47,53,54]. Table 3 summarized the effects of different pressure intensities on the denaturation of whey proteins; based on this information, the most sensitive and susceptible whey protein to HPP is β-LG, followed by IG, BSA, and α-LA. As shown, each type of whey protein has different susceptibility to pressurization and, depending on the intensity of the treatment applied, will show a different extent of denaturation and the changes in the treated milk. Different thresholds are shown that indicate the degree of denaturation of different whey proteins when subjected to distinct pressure intensities.

A report [13] on protein denaturation in whole milk subjected to 600 MPa for 5 min and compared to two pasteurization methods—LTLT (63 °C for 30 min) HTST (72 °C for 15 s)—revealed differences between samples. Bovine lactoferrin (BLF), BSA, IG, and all four different casein types remained statistically similar between the three samples; however, a significant decrease in the quantities of α-LA (6% denaturation degree) and β-LG (41 to 59% denaturation degree) was observed in the high-pressure-treated samples.

The changes and alterations in whey protein structures produced by high-pressure treatment promote some specific improvements in the functionality of milk, such as hydrophobicity, solubility, gelation, hardness, and emulsifying properties [1]. These changes may improve the quality during additional dairy product manufacturing, such as in cheese and yogurts.

#### 3.2.3. Enzymes

Specific enzymes present in milk are associated with stability loss and decreasing shelf life in raw and treated milk. The presence of psychrotrophic bacteria in milk causes the generation of heat-resistant proteolytic and lipolytic enzymes, which hydrolyze proteins and fat, causing undesirable flavors [55]. Alkaline phosphatase (ALP) and lactoperoxidase (LPO) are two of the most relevant enzymes in the milk industry, as they are traditionally used as process indicators for the efficacy and safety of thermal treatment or pasteurization [56]. Lipase is an enzyme that can naturally be present in raw milk; these enzymes degrade triglycerides into free fatty acids, monoglycerides, and diglycerides, which spoils the milk because of the undesirable aromas and flavors that are generated during this process [57].

High-pressure treatment has a dual effect on enzymes; depending on the pressure intensity, type of enzyme, and the temperature, enzymes can be activated or inhibited. Pressures lower than 350 MPa could increase the enzymatic activity because of conformational flexibility in enzymes and substrate proteins’ partial unfolding, which foments their interactions [47]. Pressures higher than 400 MPa are known to begin inactivation, which can increase alongside incrementing pressure. However, the extent of inactivation is influenced not only by the pressure level, but also by the treatment time, enzyme type, milk composition, and pH levels [7,16,47]. All of these factors play a role in the varying sensitivity of the enzymes present in milk to high pressure.

ALP resists pressure treatments up to 400 MPa; however, inactivation levels of 50% can be achieved at 500 MPa for 90 min or 600 MPa for 10 min, and a complete inactivation at 800 MPa for 8 min [54,58]. Additional indigenous enzymes—γ-glutamyl transferase (GGT), phosphohexose isomerase (PHI), and LPO—have been found to be resistant to pressures up to 400 MPa [7,54,56]. However, Munir et al. [47] summarized that GGT, PHI, and ALP are only partially inactivated at 350, 400, and 600 MPa, respectively, and completely inactivated at a pressure levels of 550, 630, and 800 MPa, respectively. Janahar et al. [59] ascertained that the lipase activity in milk samples subjected to 400 MPa during the come-up time (CUT) was statistically similar to that in untreated milk, while the sample subjected to the same pressure, but for 3 min, showed a significant increase.

A study [56] evaluated the effects of high-pressure treatments on the inactivation of the indigenous milk enzymes alkaline phosphatase, γ-glutamyl transferase (GGT), and phosphohexose isomerase (PHI). ALP presented the highest resistance to pressure, followed by GGT and PHI. A complete inactivation of GGT was observed at 600 MPa/20 °C/30 min; moreover, PHI presented an almost complete inactivation when subjected to 500 MPa/20 °C/15 min. According to these authors, ALP was the most baroresistant enzyme, as an extensive inactivation (99%) required a pressure of 650 MPa or higher. In addition, ALP is known to reactivate during cold storage in thermally treated milk; this same pattern was seen for milk samples after pressure treatment, regardless of the initial enzyme inactivation [56]. Enzymes are among the most important components of milk, as they influence shelf life and stability. As shown in Table 4, the resistance to HPP of different enzymes differs from one another, and complete inactivation can sometimes be unachievable even with the most intense treatments.

### 3.3. Carbohydrates

Milk carbohydrates are predominantly sugars—specifically, lactose, in concentrations of 4 to 5% in volume. Through conventional processing, lactose can be isomerized and degraded to lactulose; however, minimal changes have been observed when subjecting milk to high-pressure processing [1,58]. A study in which milk was subjected to pressurization in the range of 100–400 MPa for 10–60 min at 25 °C showed the lack of a Maillard reaction and no lactose isomerization [58]. In addition, Yang et al. [31] evaluated the effects of whole and skimmed milk subjected to a high-pressure treatment of 600 MPa for 5 min at an initial temperature of 23 °C; lactose concentration was determined with nuclear magnetic resonance (NMR), and was compared to untreated samples; treated and untreated skimmed milk samples had similar lactose concentrations; however, HPP-treated whole milk samples had a lower NMR intensity signal when compared to their untreated counterparts; the authors attributed this difference to a slight degradation of lactose because of the pressure treatment.

### 3.4. Minerals

As previously mentioned, the presence colloidal calcium phosphate, alongside hydrophobic interactions and hydrogen bonds, gives stability and structure to casein micelles [47]. HPP causes micelles to disaggregate and alters the mineral distribution of milk, increasing ionic calcium concentration [47,60]. The changes to calcium because of HPP alter additional physicochemical and functional properties of milks and derived dairy products, as explained in Section 4. In addition, phosphorus and magnesium are also found in casein micelles; disrupting and destabilizing these molecules causes mineral solubilization. Potassium and sodium may also present minimal changes, and can be found in the aqueous phase of milk [52]. Salt—an important component in dairy products such as cheese—undergoes changes because of HPP, causing a more efficient retention and improving its distribution in the final product [1].

A study [52] was conducted utilizing MCC and milk protein concentrates (MCPs), and a soluble mineral analysis was realized by evaluating the calcium-to-phosphorus ratio. Untreated samples had a ratio of 1:1, which increased after a treatment at 350 MPa for 15 min to 1:4; however, the 450-MPa treatment yielded a smaller ratio. The difference in results was attributed to the reassociation of α_S1_-casein, α_S2_-casein, and calcium, making them insoluble once more; nevertheless, the more intense pressure treatment still presented a higher ratio than the untreated samples.

## 4. Effects of HPP on the Physicochemical Properties of Milk

### 4.1. Effects on pH

The pH levels of milk can be altered by changes in the mineral balance caused by pressure treatment [54]. The extent of the pH changes depends on the pressure and temperature levels, the presence of microorganisms, and the milk composition [31,50,51,54]. The mechanism for pH increase by pressure has been associated with the solubilization of micellar calcium phosphate, which alters the mineral balance of milk by increasing the total amount of ionized calcium in it, and as a consequence increasing pH levels [16,54]. The composition of milk also impacts the changes to the pH levels caused by high pressure; casein micelles have a buffering capacity, so the concentration of these alters the acidity and, therefore, the pH levels after pressure treatment [51]. The presence of fat can act as a protective barrier to casein micelles, reducing their dissociation, which diminishes the changes to the milk’s pH caused by HPP [31,51]. Huppertz et al. [54] reported that, at lower temperatures, the extent of pH level shifts increases. Additionally, subjecting milk to pressures above 300 MPa causes a partial and incomplete microbial inactivation, which may impact the changes to pH levels by lowering them [51].

Yang et al. [31] observed a slight, but significant, increase in the pH of skimmed milk after a 600-MPa/5-min pressure treatment; this was not observed for whole milk or pulse-treated samples. In contrast, as seen in Table 5, Iturmendi et al. [51] observed that pH levels decreased and acidity increased in reconstituted micellar casein concentrate (MCC) treated at 300, 450, and 600 MPa for 5 min; a decrease of 0.3–0.5 units for pH levels and an increase in the acidity of 0.1–0.3 g/L were documented. Additionally, the changes to the pH levels can be irreversible during storage; nevertheless, if a rehydration of casein micelles occurs, it is possible that some changes may become reversible [31,51,54].

### 4.2. Color and Turbidity

**Figure 7:**
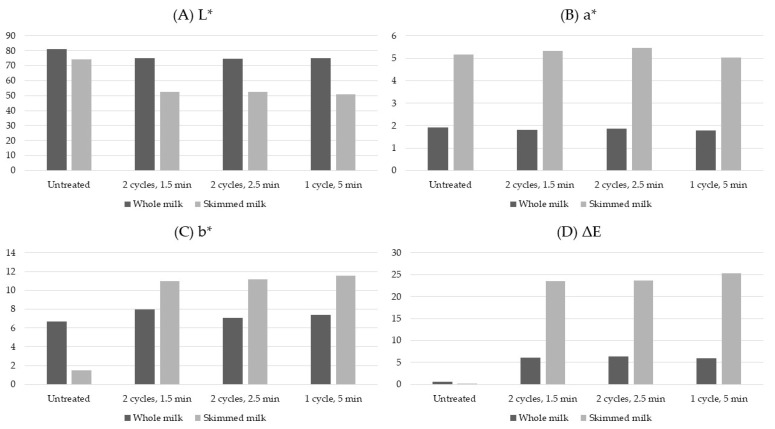
L* (**A**), a* (**B**), b* (**C**) and ΔE (**D**) color parameters of whole and skimmed milk treated at 600 MPa for different treatment times and cycles [31].

High-pressure treatment of milk and dairy products affects the color parameters and turbidity; these physicochemical properties tend to be modified [10,31,51,52,61,62]. The changes in the color and turbidity in milk have been attributed to the effects of HPP on fat globules and casein micelles, as previously explained in Section 3.1 and Section 3.2.1, respectively [10,15,31,51,52]. Milk fat globules can cause light to scatter, modifying these fat globules or altering their concentration, with a consequent impact on the color and turbidity parameters of milk [15,31,51]. Overall, skimmed milk has a decreased opacity and smaller lightness or L* values when compared to whole milk; additionally, subjecting skimmed milk to pressures that exceed 300–400 MPa also results in reduced turbidity [31]. Whole milk behaves slightly differently due to the presence of fat, which acts as a protective barrier; thus, the L* values and opacity are expected to be higher; however, subjecting whole milk to intense-pressure treatments has been shown to reduce its whiteness levels, turbidity, and opacity [10,31,51,52,62]. The a* and b* parameters of the CIE L*a*b* scale of color—which represent the green to redness and blue to yellowness color ranges, respectively—are mostly influenced by the fat and β-carotene content in milk, and high pressure may alter these values; however, lightness is the parameter that is most severely affected by HPP treatments [51]. The changes in the a* and b* values as a result of high pressure differ depending on the study and type of milk. A study on ewe milk [61] subjected to 500 MPa for 2 min yielded a statistically significant decrease in L* vales and an increase in the a* and b* values. For reconstituted MCC [51] treated at 300, 450, and 600 MPa for 5 min, an increase in the L* and b* values and a decrease in a* values was observed; additionally, after pressure treatment, L* values decreased with the increase in pressure, but the a* and b* values only showed a statistically significant increase for the most intense pressure treatment—this was attributed to the low concentration of fat (2%). In addition, Yang et al. [31] analyzed both whole and skimmed milk samples subjected to 600 MPa for up to 5 min. In this study, as seen in Figure 7, L* values decreased for both samples, b* values increased exclusively for skimmed milk, a* values remained similar for both samples, and total color difference (ΔE) increased significantly for pressure-treated skimmed milk samples because of the decreased whiteness values. These authors concluded that fat acted as a baroprotector for whole milk samples, whereas skimmed milk samples are more susceptible to high-pressure-induced color changes.

The causes of the changes in lightness levels and turbidity are associated with the changes to the casein micelles and the presence or lack of the lipid fraction in milk. As previously described in this review, the intense-pressure treatments cause water molecules to forcefully enter the casein micelles, hydrating them, liberating calcium phosphate, and causing disruption that lowers molecule size. These high-pressure-induced changes in casein micelles have been associated with the changes in color and turbidity parameters as the ability to scatter light decreases [51]. The presence of fat also affects the intensity of the changes that high pressure causes to milk’s color and turbidity. Fat protects the micelles, which helps to diminish the effect of this treatments on color parameters [31]. Additionally, as the changes caused to casein micelles by high pressure can be irreversible, the modification of color and turbidity can also be considered irreversible; because of the association of whey proteins with dissociated micelles, however, rehydration of casein micelles during storage can slightly increase L* or lightness levels [31,51].

### 4.3. Emulsion Stability

Emulsion stability refers to how the properties of size distribution, flocculation, and droplet arrangement of an emulsion change overtime [63]. Emulsion destabilization can negatively impact specific characteristics, such as organoleptic and nutritional properties, by generating changes in the physical appearance of a food product, undesirable flavors, and promoting faster nutrient degradation [63]. These changes can be attributed to the exposure of the fat fraction after emulsion destabilization, which causes it to be more susceptible to chemical reactions such as oxidation and those originating from enzymes.

As previously mentioned, HPP influences native milk proteins and those in the milk fat globule membrane (MFGM); the changes caused by this processing affect the emulsion stability of milk. Proteins have the ability to absorb both polar and non-polar components of an oil-in-water mixture, which prevents coalescence and forms an emulsion [63]. In general, low-intensity pressure treatments cause the unfolding and denaturing of proteins, so HPP can improve emulsion stability via the exposure to hydrophobic groups, leading to droplet size reduction and modifications of the protein molecular flexibility, and causing a homogeneous distribution of polar and non-polar amino acid residues, further improving the emulsifying properties [63]. In addition, protein denaturation by splitting the structure generates low-molecular-weight components, which have been shown to promote emulsion stability [63]. However, more intense and longer pressure treatments—400 MPa or higher—have been shown to negatively impact the emulsifying capacity of milk’s native proteins; this phenomenon is associated with the decrease in the solubility of whey proteins, as they tend to aggregate in the oil–water interface, reducing their emulsifying capacity [63].

The components of the MFGM help to stabilize and maintain the emulsion stability within an appropriate particle size and a dispersed phase; specifically, the protein and phospholipid content are increased, as these are adsorbed on to the membrane during processing [33]. Galazka et al. [64] studied the effects of HPP on the droplet size of emulsion solutions. Minimal changes were observed in samples treated at pressures lower than or equal to 400 MPa, whereas larger droplets were produced at 600 MPa. In the case of samples that have been previously homogenized, subjecting them to pressures in this intensity tends to increase droplet flocculation or aggregation.

### 4.4. Viscosity

Viscosity is a very important factor for milk and dairy products; traditionally, this factor can be altered during processing, or can differ between samples because of composition. Increasing the amount of milk solids—through evaporation, filtration, or other means—directly impacts viscosity; specifically, lactose and proteins are the components that most greatly impact this factor [63]. However, traditional whole and skimmed milk are usually characterized as Newtonian fluids because of their relatively low solid content, whereas concentrated milks tend to display a pseudoplastic, shear-thinning behavior [63].

Milk viscosity is altered by HPP, and the extent of the changes depends on the treatment intensity and the composition of the milk. As previously explained, the denaturation of casein micelles caused by HPP generates smaller particles and causes an increase in casein micelle hydration; both of these changes are considered the prime factors in the alteration of the viscosity of milk treated by HPP [57].

As seen in Table 6, the viscosity of traditionally thermal-processed milk was statistically similar to that of raw milk. Regarding high-pressure-treated samples, a notable increase in viscosity was presented. An additional study [49] involving 4% and 8% casein concentrates evaluated the viscosity after treatment at 300, 450, and 600 MPa for 5 min. In said study, no statistically significant differences were observed between the treated and untreated concentrates, which was attributed to the minimal changes observed in the analysis of the particle size and volume of the final products.

### 4.5. Sensory Properties

Beside extending milk’s shelf life, HPP can produce food products with a high sensory quality, as they closely resemble their natural counterparts, unlike other processed foods, as fewer chemical changes are produced [6,13,44].

High-pressure-treated milk has shown to be better evaluated in specific hedonic sensory properties and parameters than thermally pasteurized milk; aroma showed no significant difference; however, color, taste, and aftertaste were better evaluated in HPP milk [64]. Regarding the sensory analysis or evaluation of high-pressure-treated milk, it is usually compared to a heat-treated or pasteurized counterpart, and the expected results are similar or improved results in the pressurized samples. Liepa et al. [64] performed a sensory analysis comparing milk samples processed at 400 MPa for 15 min at room temperature, HTST-pasteurized milks at 78 °C for 15 to 20 s, and untreated milk, by both trained and untrained panelists. As seen in Table 7, the untrained panelists considered that the color, taste, and aftertaste of the high-pressure-treated milk samples were statistically superior to their pasteurized counterparts, while aroma received similar evaluation between the two samples. Trained panelists, on the other hand, considered the untreated samples to be the superior ones, with the high-pressure-treated milk being statistically similar in terms of taste and aftertaste, while the results for color were similar between pressurized and pasteurized samples, and the perceived aroma was statistically similar for all three samples. These authors concluded that high-pressure-treated samples presented significant differences regarding color, taste, and aftertaste, and no significant difference in terms of aroma. Additionally, while HPP milk is not able to retain the original characteristics of fresh, untreated milk, it has superior sensory properties when compared to pasteurized milk.

**Figure 8:**
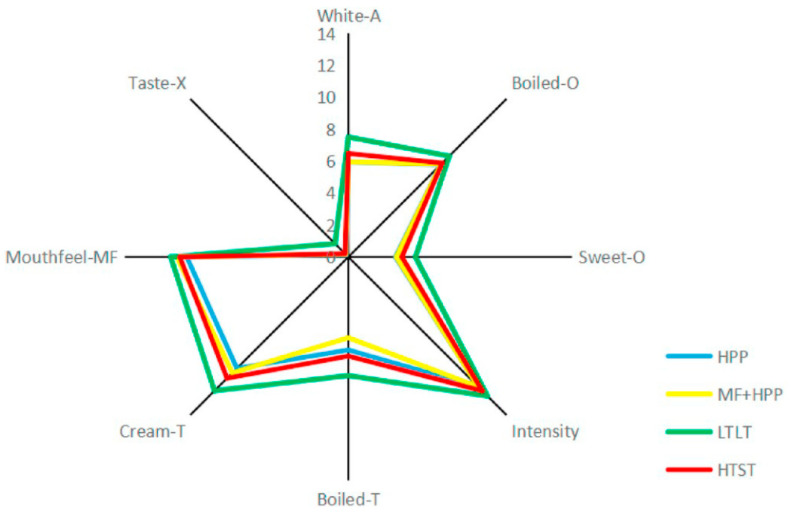
Sensory analysis comparison of LTLT (low-temperature, long-time), HTST (high-temperature, short-time), HPP (high-pressure processing), and MF + HPP (microfiltration + HPP) milk regarding appearance (A), odor (O), taste (T), intensity, mouthfeel (M), and off-taste (X). Adapted from [13]. Reproduced with permission from Guanchen Liu, Christina Carøe, Zihan Qin, Daniel M.E. Munk, Michael Crafack, Mikael A. Petersen, Lilia Ahrné, Comparative study on quality of whole milk processed by high hydrostatic pressure or thermal pasteurization treatment; published by Elsevier, 2020.

This is consistent with the findings of Liu et al. (Figure 8) [13], who evaluated and compared the sensory acceptability of heat-pasteurized and HPP-treated milk after storage for 8 days; the high-pressure-treated samples had significantly lower boiled and sweet odors, boiled and cream flavors, intensity, and white color; meanwhile, mouthfeel and taste after spitting were similar to the other samples. Overall, it has been widely accepted that high-pressure-treated milk has similar or superior sensory characteristics to those of pasteurized milk; the changes in flavor caused by heat are prevented which, in turn, causes the flavors to resemble those of natural, fresh, untreated milk, making it more acceptable for consumers.

## 5. High-Pressure-Treated Milk and Its Effects on Dairy Products

Dairy products manufactured from milk subjected to HPP have been shown to exhibit distinct benefits regarding improvements in their production, efficiency, or yield, and better sensorial and technological properties. As previously explained, HPP alters most of milk’s macronutrients—such as proteins and fats—and micronutrients (mainly minerals). These changes to milk are responsible for the alterations and modifications to different dairy product properties.

### 5.1. Cheese

Changes in cheese are the most frequently studied among dairy products regarding high pressure. The most relevant changes caused by utilizing high-pressure-treated milk are the modification of coagulation time, acceleration of ripening, increasing of yield, and modifications to physicochemical and sensory properties [1,4,7,21,54,58,66,67,68,69]. Some changes are related to the denaturation of whey proteins and the fragmentation of casein micelles—high pressure causes these two protein fractions to interact with each other. Therefore, wet-yield curd is increased as whey proteins—such as β-Lg—bond to casein micelles, causing them to not separate during coagulation; in addition, water-holding capacity and distribution are improved [1,67,68]. In addition to the improved yield and water retention properties, the change in proteins alters rennet coagulation time; however, the extent and type of changes depend on the intensity of pressure applied. Milder pressures—lower than or equal to 200 MPa—improve and reduce the time required for coagulation, as casein micelle size is reduced, which expands the surface area for the enzymatic process to be carried out more efficiently [4,7]. As the intensity of the treatment increases, rennet coagulation time increases, similarly to that of untreated milk, because more extensive whey protein denaturation causes interaction between β-lg and κ-casein which, in turn, causes the latter to be less susceptible to enzymatic action, slowing the release of the casein macropeptide [4,7,58].

Ripening is one of the final steps in the cheese manufacturing procedure; it is highly important because it dictates the final product’s quality and, therefore, is very expensive [1,67]. Reducing the ripening time for cheese manufacturing is a highly desirable; as this time-consuming step is accelerated, its costs are reduced. HPP has been shown to exert these positive effects without altering other, equally important characteristics of ripened cheese, such as quality and sensorial attributes [1,4,67]. The cheese-ripening process consists of a series of enzymatic processes that involve proteolysis, lipolysis, and glycolysis—the latter being the most important and relevant one, as it is responsible for the flavor and texture changes in the final product [4]. The influence of high pressure on cheese ripening involves alterations to enzyme structures and changes to casein micelles’ matrices which, in turn, makes treated products more susceptible to proteolysis, and enables a more efficient release of microbial enzymes due to bacterial lysis. In addition, the shifts in pH levels and modification of water distribution provide more ideal conditions for the enzymatic activity [67,68]. Today, a new perspective on the influence of HPP on cheese ripening has been considered; treatment of cheese products in order to partially or totally inactivate culture microorganisms and enzymes, slowing down the ripening process, is being considered [66,68]. The importance of preventing over-ripening is that aged cheeses can maintain specific sensory characteristics for longer, as proteolysis and lipolysis, along with the generation of undesirable volatile compounds, are retarded at pressure intensities of 600 MPa, and an extended shelf life can be achieved [66].

Finally, some disadvantages of utilizing high-pressure-treated milk in cheese manufacturing have been discussed. Color and texture changes in specific types of cheeses have been reported, specifically with cheese rinds and the internal color. When compared to traditionally processed cheese, the HPP-treated cheeses are smaller and have uneven heights in their structure; however, during the aging process, the differences between both grow smaller, and the final products are relatively similar [66]. Additionally, the color of the high-pressure cheese’s interior is modified because of the mechanisms previously explained; a loss of brightness and increase in yellowness cause the overall color difference to be greater because of the high-pressure treatment [66]. Finally, the extent of these changes is dependent on the process parameters and, more importantly, on the type of cheese being produced.

### 5.2. Yogurt

Yogurt’s rheological properties are highly affected by HPP; these changes have also been associated with an increase in quality and shelf life. During yogurt manufacturing, denatured whey proteins interact via disulfide bonds with casein micelles, followed by a fermentation process in which LAB causes a drop in pH levels, obtaining a product with altered texture and flavors [70]. The interactions between denatured whey proteins and caseins are of utmost importance because, during the fermentation step, the pH levels drop to the isoelectric point of caseins, and coalescence is avoided [71].

HPP has been shown to improve specific textural and rheological characteristics in yogurts. Pressurizing causes an increase in the viscosity of milk and yogurt immediately after treatment, and up to 60 days after storage, when compared to heat-treated samples. Syneresis, one of the most common defects in yogurts, was reduced, and the visual appearance was thicker and smoother [71,72]. Additional changes include color alterations—specifically to the L* and b* values—increased yield stress, and increased whey holding capacity [1,71,72]. Finally, lipolysis and lipid oxidation in yogurts were compared between thermally treated and pressurized samples [38]; the degree of lipolysis was similar among all different samples, and the extent of lipid oxidation was not discernable; however, the presence of malondialdehyde was detected exclusively in heat-treated yogurt samples.

### 5.3. Other Products

The HPP-induced changes to milk lipids and proteins cause distinct effects on ice creams, buttermilks, and other, similar dairy products. In general, high pressure induces fat crystallization; this causes a reduction in aging time for ice cream mixes, enhances butter ripening, and improves the whipping capacity of cream [1,73]. Additionally, water content is compressed during pressurization, lowering the freezing point of milk, which can aid ice cream manufacturing by fomenting smaller ice crystal formation, improving the texture and quality of the final product, and may extend shelf life [73]. In addition, buttermilk suffers compositional changes after high-pressure homogenization (100 MPa); smaller fat globules are observed alongside higher protein and phospholipid content [33].

## 6. Final Remarks

High-pressure processing is a technology that has been widely studied within the dairy industry for the past decades. Many researchers have pointed out its promising potential as an alternative to thermal pasteurization, based on the inactivation and reduction of raw milk’s most common microorganisms, such as *E. coli, Salmonella*, and *L. monocytogenes*. Nevertheless, limited information is available regarding the effects of high pressure on less common bacteria, such as *Staphylococcus aureus*, *Coxiella burnetii*, and *Mycobacterium*, among others. When compared to pasteurized milk, similar microbial reductions are achieved, but the extent of changes and alterations is lessened in HPP milk, as quality is not lost. Additionally, in more recent years, the focus has shifted to the effects of high-pressure processing on the physicochemical and functional properties of milk—specifically, how this treatment alters the major components of milk including proteins, lipids and minerals, and how these changes affect specific properties and characteristics. Color, turbidity, pH, emulsion stability, and rheology are some of the properties affected by these changes; however, there is an area of opportunity regarding the extent and specific causes of the alteration. Specifically, more in-depth research could be conducted to evaluate the effects of high pressure on milk fat globules, the extent of enzyme denaturation and emulsion stability in processed milk, and the interactions taking place on a molecular level. Determining the cause and extent of said changes could provide insight in order to more appropriately determine the most suitable high-pressure treatment conditions, as bacterial inactivation is not the sole factor considered.

## Figures and Tables

**Table 1:** Effects of HPP on the inactivation of microorganisms of milk.

Sample		Treatment	Microorganism	Logarithmic Reductions	Reference
Raw, whole		600 MPa, 5 min, 40 °C	Total bacterial count	3.00	[13]
			*E. coli*	3.00	
Raw, whole		350 MPa, 20 min, 10 °C	*L. monocytogenes*	0.65	[18]
		400 MPa, 10 min, 10 °C		2.10	
Raw, whole		400 MPa, 1 min, 25 °C	*E. coli*	0.85	[15]
		600 MPa, 3 min, 25°C		5.60	
		600 MPa, 5 min, 25 °C		6.80	
		400 MPa, 1 min, 25 °C	*Salmonella* spp.	1.09	
		400 MPa, 5 min, 25 °C		2.36	
		500 MPa, 5 min, 25 °C		3.28	
		600 MPa, 5 min, 25 °C		6.27	
		400 MPa, 5 min, 25 °C	*L. monocytogenes*	1.42	
		500 MPa, 5 min, 25 °C		5.48	
		600 MPa, 1 min, 25 °C		5.91	
		600 MPa, 3 min, 25 °C	TVC	3.95	
		600 MPa, 3 min, 25 °C	*Enterobacteriaceae*	Below detection limit	
		600 MPa, 3 min, 25 °C	LAB	Below detection limit	
		600 MPa, 3 min, 25 °C	*Pseudomonas* spp.	Below detection limit	
Raw, whole		600 MPa, 10 min, 25 °C	*L. monocytogenes*	6.47	[19]
		600 MPa, 10 min, 25 °C	TVC	5.09	
Pasteurized, whole	200 MPa, CUT, 25 °C		*E. coli* K12	0.50	[20]
	200 MPa, 15 min, 25 °C			1.20	
	300 MPa, CUT, 25 °C			0.50	
	300 MPa, 5 min, 25 °C			1.50	
Pasteurized, whole	500 MPa,10 min, 5 °C		*S. aureus* CECT4013	1.90	[21]
	500 MPa, 10 min, 20 °C			3.40	
	500 MPa, 10 min, 5 °C		*S. aureus* ATCC13565	1.50	
	500 MPa, 10 min, 20 °C			2.00	
UHT, whole	600 MPa, 10 min, 25 °C		*S. typhimurium*	5.10	[22]
UHT, whole	500 MPa, 10 min, 25 °C		*L. monocytogenes*	6.20	[23]
Sterile, whole	500 MPa, 5 min, 25 °C		*L. monocytogenes*	5.00	[24]
Sterile, buffer	448 MPa, 11 min, 41 °C		*L. monocytogenes*	6.00	[25]
UHT, skim	530 MPa, 30 min, 8 °C		*B. sporothermodurans* LTIS27 spores	5.00	[26]
UHT, skim	600 MPa, 15 min, 25 °C		*E. coli*	6.70	[27]

LAB = lactic acid bacteria; TVC = total mesophilic aerobic bacteria; room temperature considered as 25 °C; UHT = ultra-high temperature.

**Table 2:** Effects of HPP on casein micelle size.

Sample	Treatment	Observation	Reference
Raw skimmed milk	100–200 MPa	No observable changes in casein micelle size.	[10,39,50]
	250 MPa, 15 min, 40 °C	Approximately 25–30% increase in micelle size; reversible during storage.	
	200 MPa, 60 min, 30–40 °C		
	300 MPa, 5 min, 40 °C		
	>300 MPa, 40 °C	Approximately 50% decrease in micelle size; irreversible.	
	300–800 MPa		

**Table 3:** Effects of different pressure intensities on whey proteins [10,34,47,53,54].

Protein Fraction	Pressure (MPa)	Effect
α-LA	600	10% denaturation
	800	50% denaturation
β-LG	100	Denaturation begins
	400	70–80% denaturation
	800	90% denaturation
IG	300	Denaturation begins
	500	35% denaturation
BSA	400	Resistant; no denaturation
	600	Denaturation begins

α-LA = α-lactalbumin; β-LG = β-lactoglobulin; IG = immunoglobulin; BSA = bovine serum albumin.

**Table 4:** Effects of HPP on indigenous milk enzymes.

Enzyme	Pressure (MPa)	Effect	Reference
ALP	400	Resistant up to this pressure	[54,58]
	600	50% reduction	
	800	Complete inactivation	
LPO	400	Resistant up to this pressure	[7,54,56]
GGT	350	Partial inactivation	[47]
	400	Resistant up to this pressure	[7,54,56]
	550	Complete inactivation	[47]
	600	Complete inactivation	[56]
PHI	400	Partial inactivation	[47]
	500	Complete inactivation	[56]
	630	Complete inactivation	[47]
Lipase	400	No change in enzyme activity	[59]

ALP = alkaline phosphatase; LPO = lactoperoxidase; GGT = γ-glutamyl transferase; PHI = phosphohexose isomerase.

**Table 5:** pH and acidity of pressurized reconstituted micellar casein concentrate during storage at 10 °C [51].

Sample	Pressure (MPa)	Time	pH	Acidity (g Lactic acid/L)
4% Reconstituted MCC	Control	Day 0	7.12 ± 0.02	0.37 ± 0.01
	300		7.12 ± 0.02	0.37 ± 0.01
	450		7.21 ± 0.12	0.49 ± 0.07
	600		6.93 ± 0.05	0.64 ± 0.02
	Control	Day 7	7.15 ± 0.01	0.44 ± 0.07
	300		6.83 ± 0.02	0.66 ± 0.06
	450		7.15 ± 0.02	0.57 ± 0.04
	600		6.86 ± 0.03	0.57 ± 0.02
8% Reconstituted MCC	Control	Day 0	7.06 ± 0.00	0.82 ± 0.04
	300		7.25 ± 0.02	0.80 ± 0.03
	450		7.01 ± 0.01	1.07 ± 0.02
	600		6.75 ± 0.03	1.21 ± 0.06
	Control	Day 7	6.96 ± 0.10	1.12 ± 0.06
	300		6.71 ± 0.02	1.01 ± 0.13
	450		7.01 ± 0.07	0.97 ± 0.24
	600		6.77 ± 0.00	1.34 ± 0.26

**Table 6:** Viscosity of milk processed with different preservation methods [59].

Sample	Treatment Conditions	Treatment Conditions	Treatment Conditions	Viscosity (mPa.s)
Raw milk	Untreated	2.680 ± 0.24
Thermal-processed milk	72 °C, 15 s	2.747 ± 0.19
High-pressure-treated milk	400 MPa, 40 °C, CUT	3.053 ± 0.08
High-pressure-treated milk	400 MPa, 40 °C, 3 min	3.083 ± 0.07

**Table 7:** Sensory analysis of three different milk samples (untreated, pasteurized, and high-pressure-treated) by untrained and trained panelists utilizing a hedonic scale (1 to 5, with 5 representing the greatest enjoyment) [65].

Sensory Properties	Untrained Panelists		Trained Panelists		
	Pasteurized	High Pressure	Untreated	Pasteurized	High Pressure
Aroma	4.09 ± 0.30	4.36 ± 0.90	3.44 ± 0.72	3.12 ± 0.44	3.36 ± 0.96
Color	3.91 ± 0.60	4.63 ± 0.70	4.40 *	3.20 *	3.60 *
Taste	3.82 ± 0.70	4.36 ± 0.90	4.04 ± 0.61	3.20 *	3.64 *
Aftertaste	4.00 ± 0.70	4.50 ± 0.70	3.84 ± 0.63	3.08 *	3.64 *

* Original authors did not report the standard deviation of these results.

## Data Availability

Not applicable.

## References

[1] Chawla R., Patil G.R., Singh A.K. (2011). High hydrostatic pressure technology in dairy processing: A review. J. Food Sci. Technol..

[2] Arnold N., Yang L., Boyer R., Saunders T. (2019). How Is Pasteurization Used to Process Food?. Virgina Coop. Ext..

[3] Yang B., Shi Y., Xia X., Xi M., Wang X., Ji B., Meng J. (2012). Inactivation of foodborne pathogens in raw milk using high hydrostatic pressure. Food Control.

[4] San Martín-González M.F., Welti-Chanes J., Barbosa-Cánovas G.V. (2006). Cheese manufacture assisted by high pressure. Food Rev. Int..

[5] Yordanov D.G., Angelova G.V. (2010). High pressure processing for foods preserving. Biotechnol. Biotechnol. Equip..

[6] Alcántara-Zavala A.E., Serment-Moreno V., Velázquez-Lugo K.I., García-Alméndarez B.E., Welti-Chanes J. (2018). High pressure processing (HPP) and in-situ nisin biosynthesis by Lactococcus lactis: A hurdle approach to improve Listeria spp. inactivation in bovine milk. Rev. Mex. Ing. Química.

[7] Naik L., Sharma R., Rajput Y., Manju G. (2013). Application of High Pressure Processing Technology for Dairy Food Preservation - Future Perspective: A Review. J. Anim. Prod. Adv..

[8] Serment-Moreno V., Barbosa-Cánovas G., Torres J.A., Welti-Chanes J. (2014). High-pressure Processing: Kinetic Models for Microbial and Enzyme Inactivation. Food Eng. Rev..

[9] Carreño J.M., Gurrea M.C., Sampedro F., Carbonell J.V. (2011). Effect of high hydrostatic pressure and high-pressure homogenisation on Lactobacillus plantarum inactivation kinetics and quality parameters of mandarin juice. Eur. Food Res. Technol..

[10] Goyal A., Sharma V., Upadhyay N., Sihag M., Kaushik R. (2013). High Pressure Processing and Its Impact on Milk Proteins: A Review. Res. Rev. J. Dairy Sci. Technol..

[11] Valdez-Fragoso A., Mújica-Paz H., Welti-Chanes J., Torres J.A. (2011). Reaction Kinetics at High Pressure and Temperature: Effects on Milk Flavor Volatiles and on Chemical Compounds with Nutritional and Safety Importance in Several Foods. Food Bioprocess Technol..

[12] Cheng N., Barbano D.M., Drake M.A. (2019). Effect of pasteurization and fat, protein, casein to serum protein ratio, and milk temperature on milk beverage color and viscosity. J. Dairy Sci..

[13] Liu G., Carøe C., Qin Z., Munk D.M.E., Crafack M., Petersen M.A., Ahrné L. (2020). Comparative study on quality of whole milk processed by high hydrostatic pressure or thermal pasteurization treatment. LWT.

[14] Bogahawaththa D., Buckow R., Chandrapala J., Vasiljevic T. (2018). Comparison between thermal pasteurization and high pressure processing of bovine skim milk in relation to denaturation and immunogenicity of native milk proteins. Innov. Food Sci. Emerg. Technol..

[15] Stratakos A.C., Inguglia E.S., Linton M., Tollerton J., Murphy L., Corcionivoschi N., Koidis A., Tiwari B.K. (2019). Effect of high pressure processing on the safety, shelf life and quality of raw milk. Innov. Food Sci. Emerg. Technol..

[16] Liepa M., Zagorska J., Galoburda R. (2016). High-pressure processing as novel technology in dairy industry: A review. Res. Rural Dev..

[17] Trujillo A.J., Capellas M., Saldo J., Gervilla R., Guamis B. (2002). Applications of high-hydrostatic pressure on milk and dairy products. Innov. Food Sci. Emerg. Technol..

[18] Mussa D.M., Ramaswamy H.S., Smith J.P. (1998). High pressure (HP) destruction kinetics of Listeria monocytogenes Scott A in raw milk. Food Res. Int..

[19] Erkmen O., Dogan C. (2004). Effects of ultra high hydrostatic pressure on Listeria monocytogenes and natural flora in broth, milk and fruit juices. Int. J. Food Sci. Technol..

[20] Ramaswamy H.S., Jin H., Zhu S. (2009). Effects of fat, casein and lactose on high-pressure destruction of Escherichia coli K12 (ATCC-29055) in milk. Food Bioprod. Process..

[21] López-Pedemonte T., Roig-Sagués A.X., De Lamo S., Gervilla R., Guamis B. (2007). High hydrostatic pressure treatment applied to model cheeses made from cow’s milk inoculated with Staphylococcus aureus. Food Control.

[22] Guan D., Chen H., Hoover D.G. (2005). Inactivation of Salmonella typhimurium DT 104 in UHT whole milk by high hydrostatic pressure. Int. J. Food Microbiol..

[23] Misiou O., van Nassau T.J., Lenz C.A., Vogel R.F. (2018). The preservation of Listeria-critical foods by a combination of endolysin and high hydrostatic pressure. Int. J. Food Microbiol..

[24] Koseki S., Mizuno Y., Yamamoto K. (2008). Use of mild-heat treatment following high-pressure processing to prevent recovery of pressure-injured Listeria monocytogenes in milk. Food Microbiol..

[25] Gao Y.L., Ju X.R., Jiang H.H. (2006). Statistical analysis of inactivation of Listeria monocytogenes subjected to high hydrostatic pressure and heat in milk buffer. Appl. Microbiol. Biotechnol..

[26] Aouadhi C., Simonin H., Prévost H., de Lamballerie M., Maaroufi A., Mejri S. (2013). Inactivation of Bacillus sporothermodurans LTIS27 spores by high hydrostatic pressure and moderate heat studied by response surface methodology. LWT Food Sci. Technol..

[27] Linton M., McClements J.M.J., Patterson M.F. (2001). Inactivation of pathogenic Escherichia coli in skimmed milk using hhigh hydrostatic pressure. Innov. Food Sci. Emerg. Technol..

[28] German J.B., Dillard C.J. (2006). Composition, structure and absorption of milk lipids: A source of energy, fat-soluble nutrients and bioactive molecules. Crit. Rev. Food Sci. Nutr..

[29] USDA Food Data Central. https://fdc.nal.usda.gov/.

[30] Rodríguez-Alcalá L.M., Castro-Gómez P., Felipe X., Noriega L., Fontecha J. (2015). Effect of processing of cow milk by high pressures under conditions up to 900MPa on the composition of neutral, polar lipids and fatty acids. LWT Food Sci. Technol..

[31] Yang S., Liu G., Munk D.M.E., Qin Z., Petersen M.A., Cardoso D.R., Otte J., Ahrné L. (2020). Cycled high hydrostatic pressure processing of whole and skimmed milk: Effects on physicochemical properties. Innov. Food Sci. Emerg. Technol..

[32] Zamora A., Ferragut V., Guamis B., Trujillo A.J. (2012). Changes in the surface protein of the fat globules during ultra-high pressure homogenisation and conventional treatments of milk. Food Hydrocoll..

[33] Kiełczewska K., Ambroziak K., Krzykowska D., Aljewicz M. (2021). The effect of high-pressure homogenisation on the size of milk fat globules and MFGM composition in sweet buttermilk and milk. Int. Dairy J..

[34] Ye A., Anema S.G., Singh H. (2004). High-pressure-Induced interactions between milk fat globule membrane proteins and skim milk proteins in whole milk. J. Dairy Sci..

[35] Kiełczewska K., Jankowska A., Dąbrowska A., Wachowska M., Ziajka J. (2020). The effect of high pressure treatment on the dispersion of fat globules and the fatty acid profile of caprine milk. Int. Dairy J..

[36] Garcia-Amezquita L.E., Primo-Mora A.R., Barbosa-Cánovas G.V., Sepulveda D.R. (2009). Effect of nonthermal technologies on the native size distribution of fat globules in bovine cheese-making milk. Innov. Food Sci. Emerg. Technol..

[37] Made by Cow FAQs. https://www.madebycow.com.au/cold-pressed-raw-milk-faq.

[38] Serra M., Trujillo A.J., Pereda J., Guamis B., Ferragut V. (2008). Quantification of lipolysis and lipid oxidation during cold storage of yogurts produced from milk treated by ultra-high pressure homogenization. J. Food Eng..

[39] Huppertz T., Fox P.F., de Kruif K.G., Kelly A.L. (2006). High pressure-induced changes in bovine milk proteins: A review. Biochim. Biophys. Acta Proteins Proteomics.

[40] Patel H., Patel S. Technical Report: Understanding the Role of Dairy Proteins in Ingredient and Product Performance. https://www.thinkusadairy.org/resources-and-insights/resources-and-insights/application-and-technical-materials/technical-report-understanding-the-role-of-dairy-proteins-in-product-performance.

[41] Bhat M.Y., Dar T.A., Singh L.R. (2016). Casein Proteins: Structural and Functional Aspects. Milk Proteins Struct. Biol. Prop. Health Asp..

[42] Dalgleish D.G., Corredig M. (2012). The Structure of the Casein Micelle of Milk and Its Changes During Processing. Annu. Rev. Food Sci. Technol..

[43] Minj S., Anand S. (2020). Whey Proteins and Its Derivatives: Bioactivity, Functionality, and Current Applications. Dairy.

[44] Wijayanti H.B., Bansal N., Deeth H.C. (2014). Stability of Whey Proteins during Thermal Processing: A Review. Compr. Rev. Food Sci. Food Saf..

[45] Fox P.F., Morrissey P.A. (1981). Indigenous Enzymes of Bovine Milk. Enzym. Food Process..

[46] Otto T., Sicinski P. (2017). Cell cycle proteins as promising targets in cancer therapy. Physiol. Behav..

[47] Munir M., Nadeem M., Qureshi T.M., Leong T.S.H., Gamlath C.J., Martin G.J.O., Ashokkumar M. (2019). Effects of high pressure, microwave and ultrasound processing on proteins and enzyme activity in dairy systems—A review. Innov. Food Sci. Emerg. Technol..

[48] Hemar Y., Xu C., Wu S., Ashokkumar M. (2020). Size reduction of “reformed casein micelles” by high-power ultrasound and high hydrostatic pressure. Ultrason. Sonochem..

[49] Dalgleish D.G., Spagnuolo P.A., Douglas Goff H. (2004). A possible structure of the casein micelle based on high-resolution field-emission scanning electron microscopy. Int. Dairy J..

[50] Huppertz T., Fox P.F., Kelly A.L. (2004). High pressure treatment of bovine milk: Effects on casein micelles and whey proteins. J. Dairy Res..

[51] Iturmendi N., García A., Galarza U., Barba C., Fernández T., Maté J.I. (2020). Influence of high hydrostatic pressure treatments on the physicochemical, microbiological and rheological properties of reconstituted micellar casein concentrates. Food Hydrocoll..

[52] Cadesky L., Walkling-Ribeiro M., Kriner K.T., Karwe M.V., Moraru C.I. (2017). Structural changes induced by high-pressure processing in micellar casein and milk protein concentrates. J. Dairy Sci..

[53] Felipe X., Capellas M., Law A.J.R. (1997). Comparison of the Effects of High-Pressure Treatments and Heat Pasteurization on the Whey Proteins in Goat’s Milk. J. Agric. Food Chem..

[54] Huppertz T., Kelly A.L., Fox P.F. (2002). Effects of high pressure on constituents and properties of milk. Int. Dairy J..

[55] Tan S.F., Chin N.L., Tee T.P., Chooi S.K. (2020). Physico-Chemical Changes, Microbiological Properties, and Storage Shelf Life of Cow and Goat Milk from Industrial High-Pressure Processing. Processes.

[56] Rademacher B., Hinrichs J. (2006). Effects of high pressure treatment on indigenous enzymes in bovine milk: Reaction kinetics, inactivation and potential application. Int. Dairy J..

[57] Zhang D., Palmer J., Teh K.H., Flint S. (2020). Identification and selection of heat-stable protease and lipase-producing psychrotrophic bacteria from fresh and chilled raw milk during up to five days storage. LWT.

[58] Lopez-Fandiño R., Carrascosa A.V., Olano A. (1996). The Effects of High Pressure on Whey Protein Denaturation and Cheese-Making Properties of Raw Milk. J. Dairy Sci..

[59] Janahar J.J., Marciniak A., Balasubramaniam V.M., Jimenez-Flores R., Ting E. (2021). Effects of pressure, shear, temperature, and their interactions on selected milk quality attributes. J. Dairy Sci..

[60] Liepa M., Zagorska J., Galoburda R. (2017). Effect of high pressure processing on milk coagulation properties. Res. Rural Dev..

[61] Gervilla R., Ferragut V., Guamis B. (2001). High hydrostatic pressure effects on color and milk-fat globule of ewe’s milk. J. Food Sci..

[62] Leu M., Marciniak A., Chamberland J., Pouliot Y., Bazinet L., Doyen A. (2017). Effect of skim milk treated with high hydrostatic pressure on permeate flux and fouling during ultrafiltration. J. Dairy Sci..

[63] Gharibzahedi S.M.T., Hernández-Ortega C., Welti-Chanes J., Putnik P., Barba F.J., Mallikarjunan K., Escobedo-Avellaneda Z., Roohinejad S. (2019). High pressure processing of food-grade emulsion systems: Antimicrobial activity, and effect on the physicochemical properties. Food Hydrocoll..

[64] Galazka V.B., Dickinson E., Ledward D.A. (2000). Influence of high pressure processing on protein solutions and emulsions. Curr. Opin. Colloid Interface Sci..

[65] Liepa M., Zagorska J., Galoburda R., Straumite E., Kruma Z., Sabovics M. (2017). Sensory properties of high-pressure-treated milk. Food Balt.

[66] Nuñez M., Calzada J., del Olmo A. (2020). High pressure processing of cheese: Lights, shadows and prospects. Int. Dairy J..

[67] Chopde S.S., Deshmukh M.A., Kalyankar S.D., Changade S.P. (2014). High pressure technology for cheese processing-a review. Asian J. Dairy Food Res..

[68] Martínez-Rodríguez Y., Acosta-Muñiz C., Olivas G.I., Guerrero-Beltrán J., Rodrigo-Aliaga D., Sepúlveda D.R. (2012). High Hydrostatic Pressure Processing of Cheese. Compr. Rev. Food Sci. Food Saf..

[69] Costabel L.M., Bergamini C., Vaudagna S.R., Cuatrin A.L., Audero G., Hynes E. (2016). Effect of high-pressure treatment on hard cheese proteolysis. J. Dairy Sci..

[70] Soukoulis C., Panagiotidis P., Koureli R., Tzia C. (2007). Industrial yogurt manufacture: Monitoring of fermentation process and improvement of final product quality. J. Dairy Sci..

[71] Harte F., Luedecke L., Swanson B., Barbosa-Cánovas G.V. (2003). Low-fat set yogurt made from milk subjected to combinations of high hydrostatic pressure and thermal processing. J. Dairy Sci..

[72] Walker M.K., Farkas D.F., Loveridge V., Meunier-Goddik L. (2006). Fruit yogurt processed with high pressure. Int. J. Food Sci. Technol..

[73] Ozcan T., Akpinar Bayizit A., Yilmaz-Ersan L., Aydinol P. (2017). Effects of High-Pressure Technology on the Functional Properties of Milk and Fermented Milk Products. J. Life Sci..

